# Directing cyanobacterial photosynthesis in a cytochrome *c* oxidase mutant using a heterologous electron sink

**DOI:** 10.1093/plphys/kiac203

**Published:** 2022-05-06

**Authors:** Alejandro Torrado, Hannah M Connabeer, Annika Röttig, Nicola Pratt, Alison J Baylay, Matthew J Terry, C Mark Moore, Thomas S Bibby

**Affiliations:** Ocean and Earth Science, National Oceanography Centre, University of Southampton, Southampton SO14 3ZH, UK; Ocean and Earth Science, National Oceanography Centre, University of Southampton, Southampton SO14 3ZH, UK; Ocean and Earth Science, National Oceanography Centre, University of Southampton, Southampton SO14 3ZH, UK; Ocean and Earth Science, National Oceanography Centre, University of Southampton, Southampton SO14 3ZH, UK; Ocean and Earth Science, National Oceanography Centre, University of Southampton, Southampton SO14 3ZH, UK; School of Biological Sciences, University of Southampton, Southampton SO17 1BJ, UK; Institute for Life Sciences, University of Southampton, Southampton SO17 1BJ, UK; Ocean and Earth Science, National Oceanography Centre, University of Southampton, Southampton SO14 3ZH, UK; Institute for Life Sciences, University of Southampton, Southampton SO17 1BJ, UK; Ocean and Earth Science, National Oceanography Centre, University of Southampton, Southampton SO14 3ZH, UK; Institute for Life Sciences, University of Southampton, Southampton SO17 1BJ, UK

## Abstract

Photosynthesis holds the promise of sustainable generation of useful products using light energy. Key to realizing this potential is the ability to rationally design photosynthesis to redirect energy and reductant derived from photons to desired products. Cytochrome P450s (P450s), which catalyze a broad array of reactions, have been engineered into a variety of photosynthetic organisms, where their activity has been shown to be photosynthesis-dependent, thus acting as heterologous sinks of electrons derived from photosynthesis. Furthermore, the addition of P450s can increase the photosynthetic capacity of the host organism. In this study, we developed this technology further using a P450 (CYP1A1) expressed in the cyanobacterium *Synechococcus* sp. PCC 7002. We show that rationally engineering photosynthesis by the removal of a competing electron sink, the respiratory terminal oxidase cytochrome *c* oxidase, increased the activity of CYP1A1. We provide evidence that this enhanced CYP1A1 activity was facilitated via an increase in the flux of electrons through Photosystem I. We also conducted a transcriptomic analysis on the designed strains to gain a more holistic understanding of how the cell responds to rational engineering. We describe a complex response including changes in expression of genes involved in photosynthesis and electron transfer linked to respiration. Specifically, the expression of CYP1A1 resulted in the reduction in expression of other natural electron dissipation pathways. This study emphasizes the potential for engineering photosynthetic organisms in biotechnology but also highlights the need to consider the broader impacts on cellular metabolism of any rationally induced changes.

## Teaser

Photosynthesis in cyanobacteria can be engineered through the manipulation of both natural and artificial electron sinks.

## Introduction

Oxygenic photosynthesis, which catalyzes the light-dependent conversion of inorganic carbon (CO_2_) into “fixed” organic carbon molecules, is the major biochemical reaction facilitating life on earth (Falkowski and Isozaki, 2008). Oxygenic photosynthesis is divided into two processes. First, in the “light-dependent reactions,” light is absorbed by two chlorophyll-binding membrane-protein complexes (Photosystem II [PSII] and Photosystem I [PSI]), which act in series to generate energy in the form of ATP and reductant in the form of NADPH. Second, in the “light-independent reactions,” ATP and NADPH produced from the “light dependent reactions” are consumed during reduction of CO_2_ into organic carbon by the Calvin–Benson–Bassham cycle. Photosynthetic organisms, such as cyanobacteria, photoacclimate to the “average” irradiance to balance the capacity of the light-dependent and -independent reactions to achieve maximal rates of photosynthesis (Montgomery, 2014). However, many cyanobacteria naturally experience a highly dynamic light environment, changing rapidly with cloud cover and/or mixing in a water column (Labiosa et al., 2006). As such, cyanobacteria often absorb more light than they have the capacity to use, which is potentially damaging for the organism (photoinhibition). In order to relieve this stress, cyanobacteria can dissipate excess absorbed energy as heat, fluorescence, or via a number of “electron dissipation” pathways, which can act as safety valves to “vent” excess energy (Lea-Smith et al., 2016).

The main “electron dissipation” pathway in cyanobacteria involves the heterodimer Flavodiiron (Flv) 1,3 (Flv1–Flv3), which acts downstream of PSI (Santana-Sanchez et al., 2019). In high-light conditions, Flv1–Flv3 removes up to 60% of excess electrons and converts them into H_2_O (Allahverdiyeva et al., 2013). Other relevant alternative electron pathways related to energy dissipation are the cyclic electron flow around PSI mediated by NADH dehydrogenase-1 (NDH-1) and the heterodimer Flv2–Flv4 that is downstream of PSI (Bersanini et al., 2014; Ermakova et al., 2016; Lea-Smith et al., 2016; Santana-Sanchez et al., 2019). Furthermore, in cyanobacteria, the photosynthetic and respiratory machinery are localized in the same membrane and share some key elements such as cytochrome *b*_6_-*f*, the plastoquinone (PQ) pool and the soluble electron carriers plastocyanin and cytochrome *c*_6_ (Mullineaux, 2014). The cyanobacterial respiratory terminal oxidases (cytochrome *c* oxidase [COX] and alternative respiratory terminal oxidase [ARTO]), therefore, not only play an important role in cellular respiration, but can also act as photosynthetic “electron dissipation” pathways thus preventing photoinhibition (Shimakawa and Miyake, 2018a, 2018b).

While these diverse “electron-dissipation” pathways can be viewed as an “inefficiency” of photosynthesis (in terms of solar energy to biomass conversion), they can equally be viewed as elegant mechanisms for both minimizing photoinhibition in variable light environments and increasing the ATP:NADPH production ratio of the cell. It has been proposed that by replacing natural electron dissipation pathways (which typically result in the formation of water) with artificial heterologous electron sinks, it may be possible to instead directly drive useful chemical reactions using the “excess” reducing power of the light reactions of photosynthesis (Santos-Merino et al., 2020). Such engineered photosynthetic electron transfer chains could improve the fitness of the cell, increase tolerance to high light, and produce compounds of interest/value without compromising other biosynthetic pathways (Berepiki et al., 2016).

A promising group of candidates for use as artificial electron sinks are “cytochrome P450” (P450s) proteins. P450s are of interest in biotechnology because they are part of a widespread family of monooxygenases which have a broad range of potential substrates and carry out a variety of useful reactions (Girvan and Munro, 2016). Photosynthetic organisms are particularly good candidates for heterologous expression of P450s, as P450 activity requires membranes, oxygen, and reductant, all of which may be abundant in a photosynthetic cell but potentially limited in heterotrophic cells (Mellor et al., 2017). There are numerous examples of P450 proteins that have been successfully expressed in photosynthetic organisms, demonstrating that P450s can catalyze a range of reactions in a photosynthesis-dependent manner (Gnanasekaran et al., 2015; Berepiki et al., 2016; Wlodarczyk et al., 2016; Berepiki et al., 2018; Mellor et al., 2019; Santos-Merino et al., 2020).

One of the P450 proteins that has been widely studied is the mammalian P450 protein CYP1A1. This protein can metabolize a variety of toxins and drugs and it is well known for its capacity to degrade the herbicide Atrazine (Cornelissen et al., 2012; Kawahigashi et al., 2005). Furthermore, it can easily be assayed in vivo (Mohammadi-Bardbori, 2014), which makes it a prime candidate for use when investigating the ability of synthetic biology approaches to “rationally design” photosynthesis. CYP1A1 has previously been engineered into the cyanobacterium *Synechococcus* sp. PCC 7002 to analyze the effect of a heterologous electron sink on the physiology of the organism (Berepiki et al., 2016). In this system, CYP1A1 was shown to be powered by photosynthetically derived electrons from PSII and increased the maximum rate of photosynthesis, therefore, improving the overall photosynthetic efficiency of the cell. In addition, it was demonstrated that the activity of CYP1A1 could be increased by deleting the NdhD2 subunit of the NDH-1 complex (Berepiki et al., 2018). This deletion potentially reduced cyclic electron flow around PSI making more electrons available for CYP1A1 activity. CYP1A1 has also been expressed under an inducible promoter in the cyanobacterium *Synechococcus elongatus* PCC 7942 in combination with a sucrose exporter that acts as an additional sink after the light-independent reactions (Santos-Merino et al., 2020). This study demonstrated that the expression of both artificial electron sinks simultaneously resulted in an additive enhancement of photosynthetic efficiency. These studies show that expression of CYP1A1 in cyanobacterial membranes is a useful tool that can be manipulated to control the flow of photosynthetic electrons.

In this study, we investigated the effect of CYP1A1 expression in *Synechococcus* sp. PCC 7002 where the activity of the COX, the main respiratory terminal oxidase and a major “dissipation pathway” upstream of PSI, has been removed. Additionally, we also characterized in detail the photophysiology and transcriptomic responses of the strains in order to gain a more holistic picture of how cyanobacteria adapt to rational engineering of the photosynthetic electron flow.

## Results

### Construction of the ΔCOX mutants

**Figure 1:**
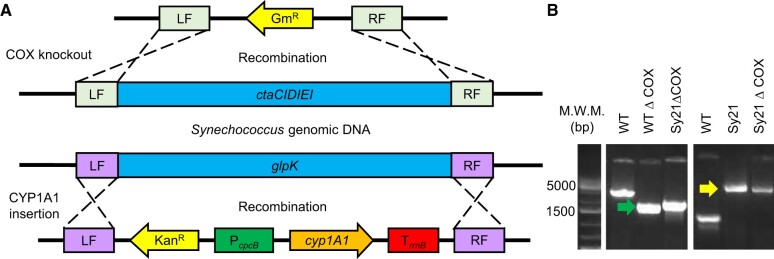
Strain design and construction. A, Cassette design for the COX knockout and CYP1A1 insertion (see Berepiki et al. [2016] for full cassette design details). B, PCR amplification of fragments between the LF and RF flanking regions of COX (green arrow) and CYP1A1 (yellow arrow) constructions. LF, left flanking region; Gm^R^, gentamicin resistance gene; RF, right flanking region; ctaCIDIEI, COX operon; glpK, glycerol kinase pseudogene; Kan^R^, kanamycin resistance gene; P_*cpcB*_, promoter of *cpcB* gene from *Synechocystis* sp. PCC 6803; *cyp1A1*, cytochrome P450 CYP1A1 from *R. norvegicus*; T_*rrnB*_, *rrnB* terminator region; and M.W.M., molecular weight marker.

*Synechococcus* sp. PCC 7002 (henceforth *Synechococcus*) has two operons that encode for two oxidase complexes, the clusters *ctaCDEI* and *ctaEDCII*. The first cluster encodes for the main *aa_3_*-type cytochrome heme-copper oxidase, COX, while the latter encodes for a *bo*-quinol oxidase, ARTO (Nomura et al., 2006; Lea-Smith et al., 2013). For the construction of the ΔCOX mutant strains, the endogenous operon was replaced with a deletion cassette consisting of a gentamicin resistance gene (Gm^R^), flanked by upstream and downstream regions of the target cluster (Figure 1A). Two strains with deletion of the COX cluster were generated (Figure 1A), the first in the wild-type (WT) background, generating the strain WTΔCOX, and the second in a background containing the CYP1A1 from *Rattus norvegicus* (described as Sy21 in previous studies; Berepiki et al., 2016, 2018), generating the strain Sy21ΔCOX. To summarize, the *CYP1A1* gene, under the P_*cpcB*_ promotor, was introduced in *Synechococcus* by replacing the pseudogene glycerol kinase (*glpK*) (SYNPCC7002_A2842) in the genome (Berepiki et al., 2016, 2018). The DNA fragment was amplified as linear DNA from a PCR (Oligonucleotides in Supplemental Table S1) and introduced into the *Synechococcus* genome by homologous recombination using its natural transformation capability. Positive candidates were selected in liquid or solid media using the relevant antibiotics, until full segregation of the gene was achieved (Figure 1B). Sy21 and Sy21ΔCOX showed similar growth rates and chlorophyll content to the WT (Supplemental Figure S1, A and B). However, WTΔCOX had a significantly lower growth rate compared with both the WT (similar to that reported by Lea-Smith et al. [2013] and Ermakova et al. [2016]) and Sy21ΔCOX (one-way ANOVA, Tukey’s post hoc test, *P*-value < 0.05). Western blot analysis of CYP1A1 confirmed that the protein was only detected in the strains Sy21 and Sy21ΔCOX and that these strains qualitatively accumulated similar amounts of protein (Supplemental Figure S1C).

### Analysis of CYP1A1 expression and activity in the ΔCOX background

**Figure 2:**
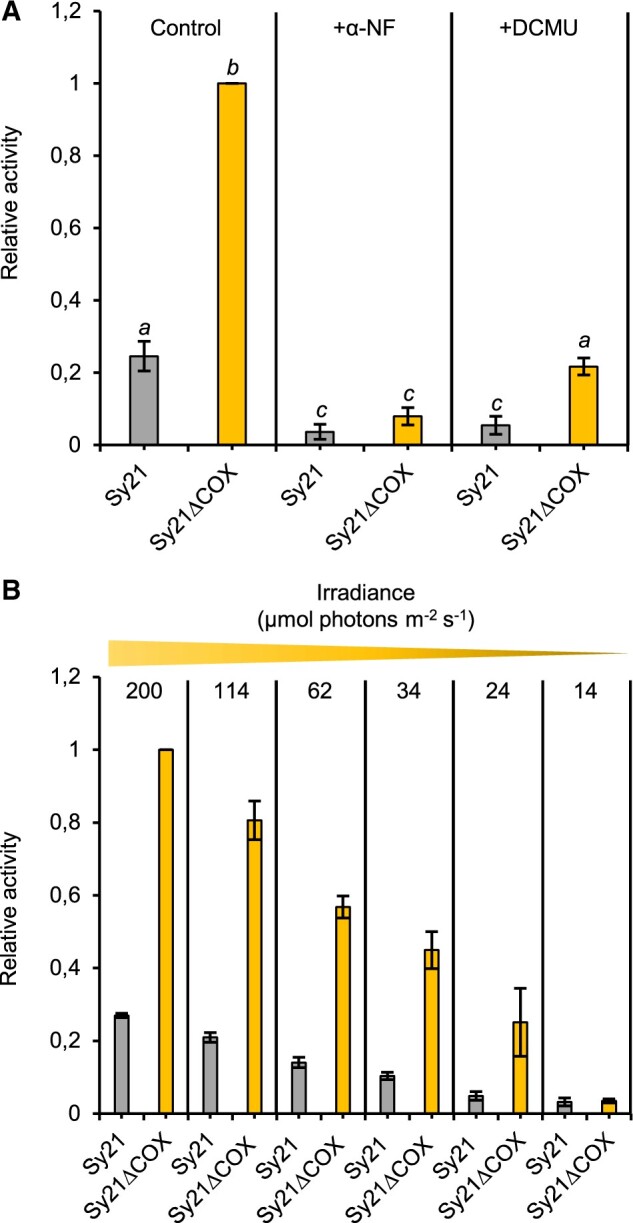
Characterization of CYP1A1 activity in the CYP1A1 expressing strains. Mean CYP1A1 activity as determined by EROD assay where the amount of fluorescent product produced is directly proportional to CYP1A1 activity. A, Characterization in the presence of the P450 inhibitor α-NF and PSII inhibitor DCMU (+DCMU). B, Characterization during a light gradient of decreasing irradiance, ranging from standard light growing conditions (200 µmol photons m^−2^ s^−1^) to reduced light conditions (14 µmol photons m^−2^ s^−1^). Different letters indicate significant difference between strains (one-way ANOVA, Tukey’s post hoc test, *P*-value < 0.05). Each value is the mean of three independent replicates, error bars represent standard deviation of the mean.

To analyze the effect of the removal of the COX operon on the activity of the heterologous sink CYP1A1, its activity was measured in Sy21 and Sy21ΔCOX in vivo, via an ethoxyresorufin O-deethylation (EROD) assay. This assay, performed in the light under standard growing conditions (see “Materials and methods”), measured the activity of CYP1A1 by monitoring the CYP1A1 dependent conversion of 7-ethoxyresorufin to the fluorescent product resorufin. In all assays, WT and WTΔCOX showed no EROD activity (Supplemental Table S2). Sy21ΔCOX had around 400% more activity under our experimental conditions compared with the parent Sy21 strain (Figure 2A). To assess whether EROD activity was driven specifically by CYP1A1, we added the CYP1A1 inhibitor α-naphthoflavone (α-NF; Figure 2A). Sy21 and Sy21ΔCOX showed a drastic reduction in EROD activity in the presence of α-NF, similar to that reported in the Sy21 parent strain by Berepiki et al. (2016). In order to demonstrate that the electrons driving CYP1A1 activity were derived directly from photosynthetic activity, the PSII inhibitor 3-(3,4-dichlorophenyl)-1,1-dimethylurea (DCMU) was added, resulting in a dramatic decrease in CYP1A1 activity in both strains. Finally, CYP1A1 activity was shown to scale with irradiance (Figure 2B) where EROD activity was measured over a light gradient.

### Electron transport rate through PSII

**Figure 3:**
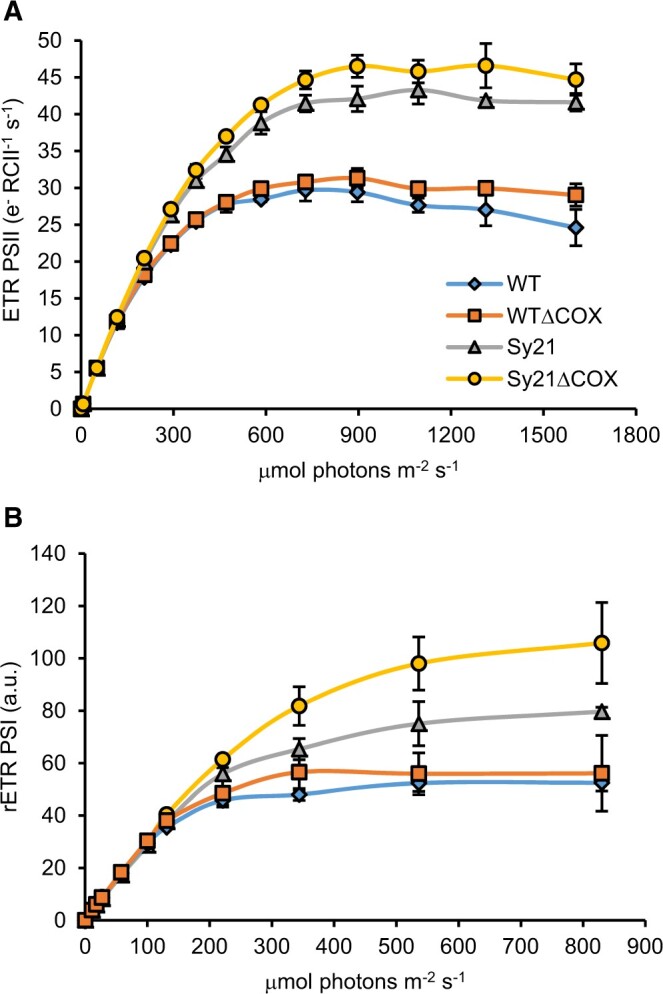
RLC measurements of PSII and PSI. RLCs describing the number of electrons produced at PSII (A) and PSI (B) at increasing measured irradiance. Plotted RLCs are the average produced from three independent replicates; error bars represent standard deviations from the mean.

To assess the impact of CYP1A1 expression on the photosynthetic capacity of the strains, the photophysiology of PSII and PSI was measured using established biophysical techniques. The electron transport rate through PSII (ETR_PSII_) was measured using Fast Repetition Rate fluorometry (FRRf). The results showed a significant (one-way ANOVA, Tukey’s post hoc test, *P* < 0.05) increase in maximum ETR (maxETR_PSII_) in Sy21 compared with WT (50%), as observed previously (Berepiki et al., 2016; Figure 3A andTable 1). Furthermore, Sy21ΔCOX showed a relatively small increase in maxETR_PSII_ compared with Sy21 (64% larger than WT), while WTΔCOX had a similar maxETR_PSII_ compared with WT (Table 1). The saturating light irradiance (E_k_), increased by ∼56% in both Sy21 and Sy21ΔCOX compared with WT (Table 1). ETR_PSII_ was also measured on all strains in the presence of the CYP1A1 inhibitor (Supplemental Figure S2A). The results showed that in the presence of the inhibitor, the ETR_PSII_ of Sy21 and Sy21ΔCOX reverted to WT levels.

**Table 1.** Photophysiological characterization of the studied strains

Strain	PSII max ETR (e^-^ RCII^−1^ s^−1^)	PSII E_k_ (µmol photons m^−2^ s^−1^)	PSI max ETR (rPSI)	PSI E_k_ (µmol photons m^−2^ s^−1^)	*F*_v_/*F*_m_
WT	28	259	51	157	0.551
WTΔCOX	30	293^*^	56	166	0.545
Sy21	42^*^	403^*^	78^*^	256^*^	0.549
Sy21ΔCOX	46^**^	407^*^	106^**^	321^**^	0.523^*^

The MaxETR of PSII/PSI was extracted from the respective RLCs by extrapolating the point of the curve where it saturates on the *Y*-axis. The saturating irradiance (E_k_) of PSII/PSI was extracted from the respective RLCs by extrapolating the point of the curve where it saturates on the *X*-axis. The *F*_v_/*F*_m_ was calculated by extrapolating the *F*_0_ and *F*_m_ from the induction recuperation curves generated from Dual-PAM. All values represent the mean of three independent replicates. Single asterisk indicates a significant difference to the WT and double asterisk indicates a significant difference to the Sy21 single mutant (one-way ANOVA, Tukey’s post hoc test, *P* < 0.05).

### Relative ETR through PSI

The characterization of the ETR through PSI (rETR_PSI_) was performed using a Dual-PAM 100. WTΔCOX did not show a significant difference in rETR_PSI_ compared with WT (Figure 3B). However, both strains expressing CYP1A1 showed a significant increase in rETR_PSI_ compared with WT. Similar to the results in Berepiki et al. (2018), Sy21 showed a 50% increase in max rETR_PSI_ compared with WT, while Sy21ΔCOX showed a 107% increase in rETR_PSI_ compared with WT (Figure 3B andTable 1). The E_k_ value followed a similar trend, with a 63% and 104% increase in Sy21 and Sy21ΔCOX, respectively, compared with WT (Table 1). rETR_PSI_ was also measured in the presence of the CYP1A1 inhibitor (Supplemental Figure S2B). The results demonstrated that in the presence of the inhibitor, the rETR_PSI_ of Sy21 and Sy21ΔCOX returned to WT (no inhibitor) level.

### PQ reduction and P700^+^ oxidation kinetics

**Figure 4:**
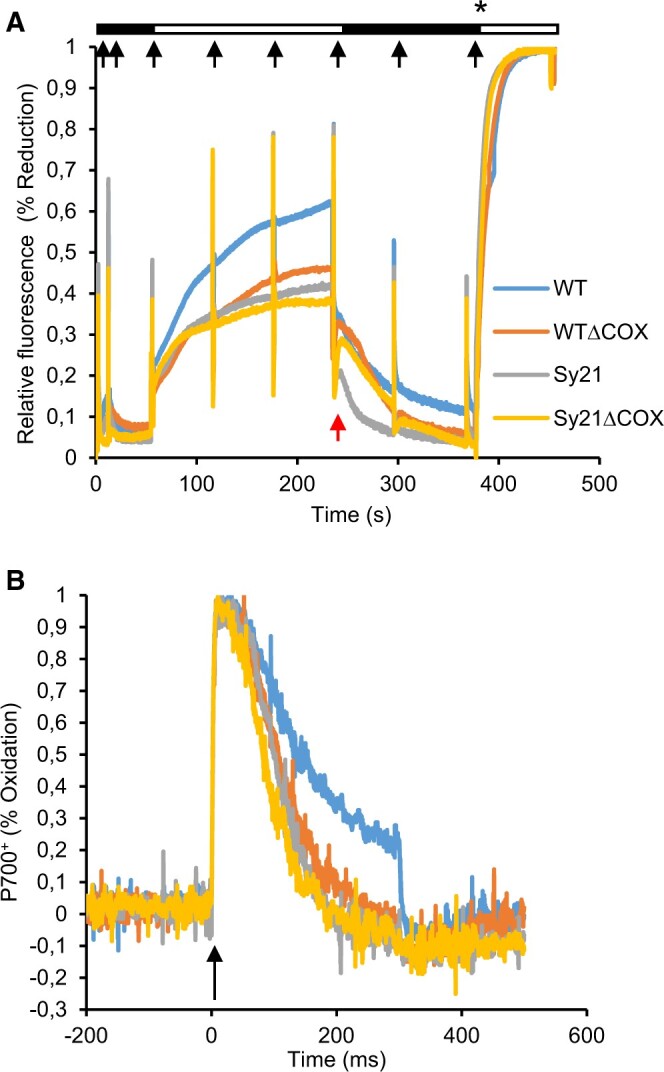
Photophysiological characterization of PSII and PSI. A, Induction recuperation curve describing the oxidation and subsequent re-reduction of the PQ pool derived using Dual-PAM 100. Black boxes represent periods of dark while white boxes represent illumination period with actinic light of 200 µmol photons m^−2^ s^−1^ (growth irradiance). Red arrow indicates the postillumination transition where the actinic light was switched off. Black arrows represent discrete saturating pulses of light (10,000 µmol photons m^−2^ s^−1^). Asterisk indicates the moment of addition of DCMU for achieving the absolute reduction of the PQ Pool. B, P700^+^ oxidation kinetics describing the speed of oxidation and subsequent re-reduction of PSI. The black arrow indicates the pulse of saturating irradiance (10,000 µmol photons m^−2^ s^−1^) that triggers the experiment. Curves are the average produced from three independent replicates.

The PQ pool reduction state was determined by induction-recovery traces using a Dual-PAM 100. This method is commonly used in plants and had to be adapted to cyanobacteria following the protocol described by Ogawa et al. (2017) with several changes (see “Materials and methods”). Due to potential interference of phycobilins, results are presented as relative values (Ogawa et al., 2017). The results (Figure 4A) indicate that WT exhibited a typical trace of oxidation/reduction, where the reduction during the first part of the trace (actinic light phase) is about 60% of the maximum (achieved after the addition of DCMU). However, the reduction during the same actinic light period was 42%, 38%, and 35% of the maximum for WTΔCOX, Sy21, and Sy21ΔCOX, respectively. At the point where the actinic light was switched off (240 s, marked by a red arrow), the capacity of re-oxidation of the PQ pool was lower in the strains lacking COX compared with the WT and Sy21 strains. The last part of the trace was used to calculate *F*_m_ (after the addition of DCMU) and this value was used to estimate *F*_v_/*F*_m_. We found no significant differences in *F*_v_/*F*_m_ between strains except for Sy21ΔCOX, which was slightly lower than in the WT (Table 1).

The oxidation/reduction kinetics of PSI were assessed by running fast kinetic traces using the Dual-PAM 100. These traces were recorded in a timeframe of 700 ms, there was an excitation pulse at time zero to oxidize the P700^+^ reaction center, followed by the measurement of the rapid re-reduction of P700^+^ through the arrival of electrons from PSII (Figure 4B). The linear part of the traces (50–100 ms) was used to calculate the re-reduction rate. The traces show that all the engineered strains had faster P700^+^ re-reduction kinetics than the WT. Specifically, we saw a 20% and 23% faster re-reduction rate in WTΔCOX and Sy21, respectively, compared with the WT, while Sy21ΔCOX had a 47% faster re-reduction rate in comparison to WT.

### Transcriptomic analysis of the mutant strains

**Figure 5:**
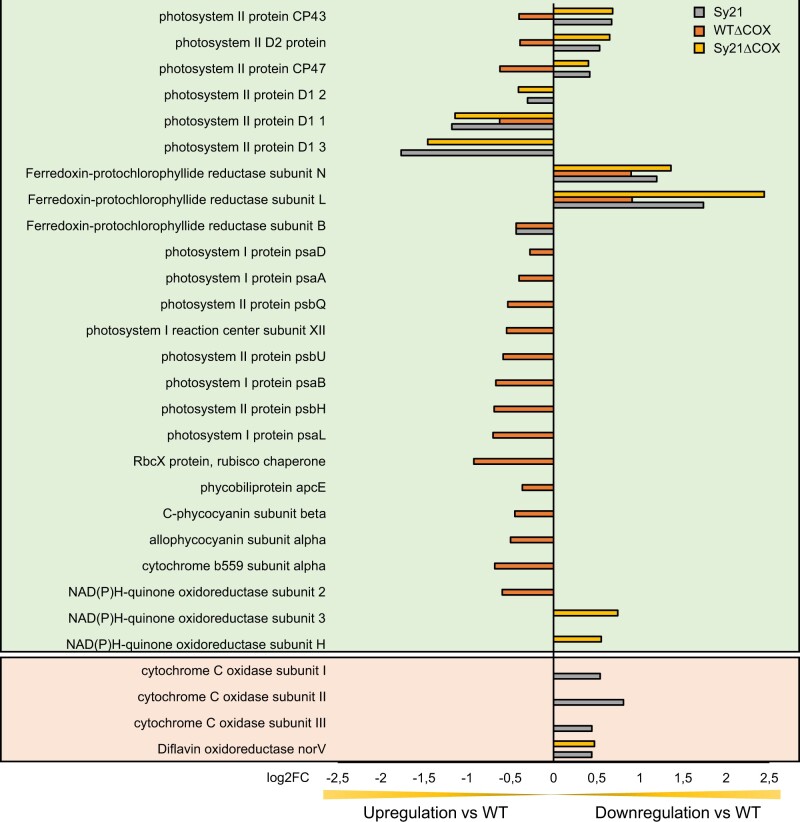
Differential regulation of genes related to photosynthesis identified from the GO enrichment analysis. The genes that are displayed in the green background were identified from the GO enrichment analysis (Supplemental Figure S5) and are complemented by other differentially regulated genes, displayed in the peach background, that are known to be potential sinks of electrons derived from photosynthesis. Bars represent differentially regulated genes of ΔCOX (orange), Sy21 (gray), and Sy21ΔCOX (yellow) in comparison to WT. Positive/negative log2FC represents genes downregulated/upregulated in the engineered strain in comparison with the WT control condition. GO enrichment analysis was constructed using the output of DESeq2 (three replicates for each condition).

To gain a more holistic understanding of how the expression of an artificial electron sink influences the molecular responses of the cyanobacterium, we conducted a transcriptomic analysis using an Illumina MiSeq benchtop sequencing platform (Illumina). The analysis was carried out on mRNA isolated from the same cultures used for physiological characterization and generated a total of 23.53 M reads. A PCA plot derived from the different transcriptomes (Supplemental Figure S3) showed a clear clustering of the replicates without overlapping of strains. In PC1 (accounting for 49.74% of the variation), we found two discrete clusters formed by WT/WTΔCOX and Sy21/Sy21ΔCOX pairs. In PC2 (accounting for 11.69% of the variation), we also found two independent clusters; however, these were formed by WT/Sy21 and WTΔCOX/Sy21ΔCOX. A Venn diagram summarizes the total number of differentially regulated genes detected in each strain in comparison to WT and the number of differentially regulated genes that are between each strain (Supplemental Figure S4A). Two further Venn diagrams describe genes that are upregulated in the strains compared with the WT (Supplemental Figure S4B) and genes that are downregulated compared with the WT (Supplemental Figure S4C). A GO enrichment analysis of the transcriptomes was performed (Supplemental Figure S5) to identify cellular functions disproportionately represented by significantly regulated genes. This analysis identified the cellular functions impacted by the expression of CYP1A1 and/or the removal of COX compared with WT. Photosynthetic and photosynthesis-related categories were identified as disproportionately regulated groups in all the studied strains. As such, we present data of photosynthetic genes that are differentially regulated in our engineered strains (Figure 5), complemented with a subset of differentially regulated genes known to be potential sinks for photosynthetic electrons.

## Discussion

### Increasing the flux of photosynthetic electrons to heterologously expressed CYP1A1

**Figure 6:**
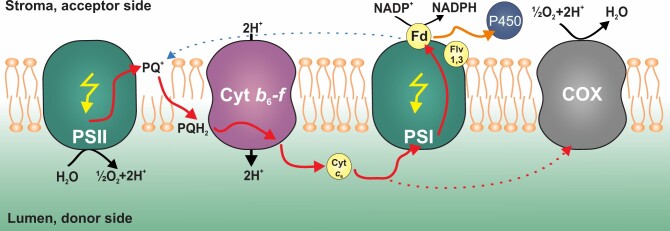
Proposed model of photosynthetic electron flow in engineered photosynthetic cell lines. PSII/PSI, photosystem II&I; PQ, plastoquinone; PQH_2_, plastoquinol; Cyt *b_6_f*, cytochrome *b_6_f*; Cyt *c*_6_, cytochrome *c_6_*; Fd, ferredoxin; Flv1,3, Flavodiiron 1,3; P450, cytochrome P450 (CYP1A1); and COX, *aa*_3_-type cytochrome-c oxidase. Red solid arrows indicate electrons of photosynthetic linear electron flow while red dotted arrow indicates electrons directed to respiration; black arrows indicate transport of H^+^ ions over the thylakoid membrane; yellow arrows represent photons; blue dotted arrow represents cyclic electron transport; and orange arrow represents the alternative electron flow toward CYP1A1.

The expression of heterologous sinks that use photosynthetically derived electrons in cyanobacteria has been demonstrated as a potential method to drive useful chemical reactions (Berepiki et al., 2016, 2018; Mellor et al., 2019; Santos-Merino et al., 2020). Heterologously expressed P450s proteins, specifically CYP1A1 (which acts after PSI), have been shown to increase photosynthetic efficiency by increasing the maximum rate of electron transfer through PSII (Berepiki et al., 2016). Manipulation of the photosynthetic electron transport chain has been proven to increase the flux of electrons to CYP1A1 (Berepiki et al., 2018). CYP1A1 has also been shown to have a positive additive effect on photosynthetic efficiency when combined with a heterologously expressed sucrose exporter (Santos-Merino et al., 2020). In this study, we constructed a rationally engineered cyanobacterial system bearing CYP1A1 that lacks the alternative natural “dissipation pathway” COX (Figure 6).

We confirmed that the activity of CYP1A1 in all engineered strains is dependent on light and driven by electrons derived from photosynthesis. Importantly, the measured CYP1A1 activity is significantly higher when the natural electron sink (the respiratory terminal oxidase COX) is removed (Figure 2). We propose that the increase in activity can be attributed to the removal of the COX, as electrons that would normally go to the COX (red dashed arrow, Figure 6) may instead be redirected to CYP1A1 via PSI (orange arrow, Figure 6). In support of this, we confirm both strains expressing CYP1A1 have similar growth rates and amounts of the CYP1A1 protein, implying that the rate of supply of electrons to CYP1A1 has been increased in the Sy21ΔCOX strain (Supplemental Figure S1). Interestingly, the Sy21ΔCOX strain had a significantly higher growth rate than the WTΔCOX strain (Supplemental Figure S1A). This improvement in growth rate could represent an advantage for its possible use in biotechnology, as the cyanobacteria will likely keep CYP1A1 to maintain its fitness.

### Removal of COX increases electron flow through PSI to CYP1A1

In order to determine how photosynthetic electrons were being differentially targeted to CYP1A1 in Sy21 and Sy21ΔCOX, the physiology of PSII and PSI was measured. For both strains, the maxETR_PSII_ and PSII E_k_ were higher than in the WT (Figure 3A). This implies that the presence of the new electron sink (CYP1A1) increased the irradiance at which photosynthesis was saturated, effectively increasing the efficiency of photosynthesis (Berepiki et al., 2016). The removal of the COX did not result in significant changes in maxETR_PSII_ in either the WT or Sy21 background, which suggests that the increased CYP1A1 activity must be due to differences downstream of PSII. Accordingly, we assessed PSI physiology and found that Sy21 had a higher maxETR_PSI_ than WT (as shown by Berepiki et al. [2018]) and that Sy21ΔCOX maxETR_PSI_ was even higher than in Sy21. This implies that the increased CYP1A1 activity in Sy21ΔCOX (compared with Sy21) was due to more electrons being transferred through PSI (Figure 3B). These results are consistent with the removal of COX, which normally competes with PSI for electrons from cytochrome *c*_6_ (Figure 6).

To further characterize the photosynthetic physiology of the studied strains, the PQ pool dynamics and P700^+^ fast kinetic traces were measured (Figure 4). The PQ pool redox kinetics in Sy21 behaved differently from the WT strain (Figure 4A). During the actinic light illumination phase, Sy21 had a more oxidized PQ Pool (in comparison with WT). This “overoxidation” can potentially be accounted for as CYP1A1 acts as a new electron sink downstream of PSI, resulting in PSI becoming more oxidized and therefore allowing for an increased flux of electrons from the PQ pool. However, in the postillumination period (after the actinic light is turned off), Sy21 had a proportionally similar re-oxidation kinetic to WT. This result was expected, since during the “dark” phase of the trace, the re-oxidation of the PQ pool is mediated by electron sinks that are not regulated by light. Therefore Sy21, containing CYP1A1 which requires electrons generated from light (Figure 2B), had a similar re-oxidation of the PQ pool as WT. This contrasts with both mutants that lack COX. Both WTΔCOX and Sy21ΔCOX had a 31% and 34% lower capacity for re-oxidation of the PQ pool compared with the WT and Sy21. This decrease can be attributed to the absence of COX, which is active in the dark; we, therefore, propose that the removal of COX results in delayed re-oxidation of the PQ pool in the dark (Figure 4A).

The P700^+^ fast kinetics revealed information on the rate at which electrons arrived at PSI. All engineered strains showed faster P700^+^ re-reduction rates compared with WT (Figure 4B), the fastest of which was Sy21ΔCOX (47% faster compared with WT). This response is intuitive for strains lacking COX, where PSI is expected to be a more substantial sink for electrons originating from PSII. However, it is a surprising response in Sy21, where the only difference with the WT (CYP1A1 protein) is downstream of PSI and therefore it is not expected to affect the arrival rate of electrons to PSI. This could potentially reflect the increased capacity of Sy21 to process electrons through PSI or reflect a broader reorganization of the photosynthetic electron transfer chain when CYP1A1 is expressed (see transcriptomic discussion below; Figure 5).

We, therefore, conclude that the CYP1A1 insertion in a ΔCOX background improves the overall photosynthetic capacity and consequentially the growth rate (compared with the WTΔCOX). While there was no improvement in the Sy21ΔCOX growth rate compared with both the WT and Sy21, the capacity for linear electron transport was improved. This improvement was mainly due to an increase in the PSI ETR (Figure 3B), resulting in the increase in CYP1A1 activity (Figure 2A). In Sy21ΔCOX, we propose: (1) there may be an increased number of respiratory derived electrons being transferred toward CYP1A1 (via PSI) or (2) that when photosynthesis is saturated, COX acts as an electron dissipation pathway such that in Sy21ΔCOX the electrons that would otherwise have been dissipated by COX are now transferred to the engineered CYP1A1. Given that the addition of DCMU, which blocks electron flow from PSII (Figure 2A), significantly reduced CYP1A1 activity in Sy21ΔCOX, it is likely that the majority of the additional CYP1A1 activity is explained by COX acting as an electron dissipation pathway.

### Transcriptional response of cells with manipulated photosynthetic electron flow pathways

While rational engineering of biological processes such as photosynthesis can seem intuitive, the organism may have a complex response to engineered changes. This response may support the designed phenotype or may act to minimize the impact of the induced change. As such, we conducted a transcriptomic analysis of all strains to determine the broader impact of CYP1A1 insertion and the removal of COX. All mutant strains presented distinct clusters from the WT in the PCA analysis (Supplemental Figure S3). This indicates that the induced changes led to multiple impacts on gene expression in the strains, where the introduction of CYP1A1 was the major factor driving changes in the transcriptome (PC1 49.74% variance). A GO-term enrichment analysis identified a number of cellular functions impacted at the gene expression level between strains (Supplemental Figure S5). These highlighted processes include protein maturation, pentose phosphate, glucose metabolism, and photosynthesis-related processes. Among these photosynthetic processes, we identified all the differentially regulated genes compared with the WT (Figure 5). These results may provide a molecular understanding of the measured photophysiology described above. The removal of COX resulted in an upregulation of a high number of the genes related to photosynthesis including subunits of both PSII and PSI, whereas the insertion of CYP1A1 resulted in the differential expression of fewer photosynthetic genes overall and specifically genes related to PSII. Notably in Sy21 and Sy21ΔCOX, we observed an upregulation of genes encoding for the alternative versions of the D1 subunit of PSII, which may be indicative of a higher turnover rate of this core protein related to the measured increased ETR_PSII_ in these strains (Wu et al., 2011). Interestingly, Sy21ΔCOX had an overall photosynthetic gene expression profile that is more similar to Sy21, suggesting that the presence of CYP1A1 is more significant in the transcriptomic profiles in general than the loss of COX. However, the absence of COX affected the expression of more photosynthetic genes in comparison to the addition of CYP1A1. Despite these changes in genes encoding PS proteins between the strains, they seemed only to have a moderate impact on the amount of PSII and PSI proteins in the strains (Supplemental Figure S6).

We observed an interesting response in natural “alternative electron flow” pathways in the Sy21 strain where the natural COX is still present (Figure 5). We found evidence for downregulation of the COX subunits as well as for a gene involved in the formation of the Flv complex (the flavin oxidoreductase Flv1). This may be a response of CYP1A1 functioning as a new dissipation pathway, resulting in the cyanobacteria suppressing the expression of natural AEF pathways. Interestingly, the lower expression of COX in the Sy21 strain may explain the result in Figure 4B, where the Sy21 strain has a similar response to strains in which COX has been removed. Altogether, these findings indicate a broad cellular impact of rational engineering of a heterologous photosynthetic electron sink, which may have impacts on diverse cellular processes, such as respiration and dissipation pathways, and should be considered when developing this kind of approach.

In summary, we introduced a heterologous electron sink (CYP1A1) and removed a natural electron dissipation pathway (COX) which resulted in (1) enhanced photosynthetic performance of PSI that resulted in (2) dramatically increased CYP1A1 activity. While these rational changes had little impact on the overall growth rate, a physiological and transcriptomic analysis of engineered strains revealed complex responses at both a molecular and whole organism scale. This study illustrates that rational engineering of photosynthesis to power useful reactions is possible and provides a platform that could be developed for more specific and sustainable applications. This work also demonstrates that the impacts of rational design should be considered with a more holistic approach to analyzing host and product interactions.

## Materials and methods

### Strain construction

All *CYP1A1* expressing strains were generated as per the method presented by Berepiki et al. (2016), with minor changes. Briefly, the *CYP1A1* gene was introduced by replacing the pseudogene *glpK* (SYNPCC7002_A2842), under the P_*cpcB*_ promotor. Strains lacking the main cytochrome oxidase (*ctaCDEI*) were generated by homologous recombination, using primers for the amplification of DNA fragments flanking the region of the COX gene and the antibiotic gentamicin gene (Figure 1 and Supplemental Table S1). Transformation of *Synechococcus* strains followed the same method presented by Berepiki et al. (2018), with the appropriate combination of antibiotics.

### Culturing conditions

WT and engineered strains of *Synechococcus* were grown in liquid A^+^ medium (containing 1  g L^−1^ sodium nitrate). Media was supplemented with 50 µg mL^−1^ kanamycin or 50 µg mL^−1^ gentamicin where appropriate. Solid A^+^ media was prepared as above with the addition of 1% (w/v) agarose (Difco Bacto Agar, Fisher Scientific, New Hampshire, USA) and 1 mM sodium thiosulfate. For physiological studies, cultures were grown in 50 mL of liquid A^+^ media with air bubbling, at 30°C under continuous white LED illumination at 200 µmol photons m^−2^ s^−1^ in a Multicultivator growth chamber (PSI Instruments, Drásov, Czech Republic). These conditions are referred to as standard growth conditions. Cultures were seeded at 0.2 OD_730_ nm and all physiological measurements were made when the cultures were in the early exponential phase (0.5–0.6 OD_730_ nm). After samples were collected for physiological analysis, the remaining culture was centrifuged at 4,000 *g* for 10 min at room temperature, snap frozen with liquid nitrogen, and stored at −80°C for protein and gene expression analysis.

### Growth rate and chlorophyll a measurements

Cell growth was monitored with a UV–Vis spectrophotometer (Jenway 7315 spectrophotometer, Staffordshire, UK) by recording the OD_730_ nm every 24 h.

Chlorophyll *a* was measured according to the method developed by Mackinney (1941). Briefly, 1 mL of exponential phase cultures (0.5–0.6 OD_730_ nm) was centrifuged at 12,000 *g* for 5 min at 4°C. The supernatant was discarded and the pellet was resuspended in 1 mL of pure methanol. The suspension was vortexed for 1 min and centrifuged again at 12,000 *g* for 5 min at 4°C. The resulting supernatant was monitored at OD_665_ nm and the concentration was calculated using the molar extinction coefficient of chlorophyll *a* in methanol of 74.46 mg^−1^ mL^−1^ cm^−1^.

### EROD assay

CYP1A1 activity was measured using the EROD assay and adapted for high-throughput microplates. The assay is based on the degradation of the substrate ethoxyresorufin into the fluorescent compound resorufin (Mohammadi-Bardbori, 2014). Cells were collected from exponentially growing cultures and normalized to 0.1 OD_730_ nm with A^+^ medium. An aliquot of 100 µL of the normalized cell culture was dispensed in triplicate into a black 96-well microplate (Greiner Bio-One, Kremsmünster, Austria). The assay was commenced with the addition of 100 µL of 5 µM 7-ethoxyresorufin prepared in A^+^ medium. The plate was incubated for 1 h under standard growth conditions with gentle shaking. The fluorescent product of the assay, resorufin, was measured on a microplate reader (excitation 544 nm, emission 590 nm; Fluostar Optima by BMG Labtech, Ortenberg, Germany).

### Photophysiology—FRRf

Photophysiological characteristics of PSII were measured on a FastTracka Mk II sensor integrated with a FastAct Laboratory system (Chelsea Technologies, West Molesey, UK) using FRRf techniques. Samples were collected from exponentially growing cultures and normalized to 0.1 OD_730_ nm with A^+^ media. The samples were dark acclimated for 30 min prior to analysis. Where appropriate, 10 µM of αNF, prepared in dimethyl sulfoxide (DMSO), was used for inhibitory experiments. Fluorescence transients were measured by excitation with blue light LEDs in order to excite chlorophyll *a*. A saturating sequence of 200 × 1 µs flashlets at a 2-µs repetition rate was used. The light response of photosynthetic electron transport was determined by measuring fluorescence parameters over an imposed actinic light gradient (Rapid light curves [RLCs]). The RLC consisted of 15 steps, ranging from 0 to 1,600 µmol photons m^−2^ s^−1^. The fluorescent transients were used to calculate the ETR from PSII. This was estimated using the equation:

$$\text{ETR}=\sigma_{\text{PSII}}\left(F_{q}^{\prime }/F_{m}^{\prime }\right)/\left(F_{v}/F_{m}\right)(E),$$

where *F*_q_′/*F*_m_′ is the ratio of variable fluorescence to maximal fluorescence measured under actinic light (*E*), *F*_v_/*F*_m_ is the ratio of variable to maximal fluorescence, and *σ*_PSII_ is the functional absorption cross section of PSII (Berepiki et al., 2018). The light response of the ETR was subsequently fitted to a standard model to derive the maximum ETR from PSII and the saturating light intensity (PSII E_k_).

### Photophysiology—PAM

Photophysiological characteristics of PSI were measured using a Dual-PAM 100 instrument (Heinz Walz, Effeltrich, Germany) with the DUAL-DR measuring head (specifically designed for cyanobacterial measurements). Cells were collected by centrifugation and resuspended in fresh A^+^ medium to 3–4 OD_730_ nm. Samples were acclimated in the dark for 30 min and then transferred to a quartz cuvette for analysis. Where appropriate, 10 µM of α-NF, prepared in DMSO, was used for inhibitory experiments. The ETR to PSI was determined by investigating the change in absorbance at 830 nm compared with the absorbance at 870 nm of the PSI primary donor P700^+^. Light curves of PSI electron transport were generated using a sequence of 11 steps of 30 s ranging from 0 to 830 µmol photons m^−2^ s^−1^, using the values calculated by Dual-PAM software for the ETR. Similar to PSII, the light response of the ETR was subsequently fitted to a standard model to derive the maximum ETR from PSI and the saturating light intensity (PSI E_k_). To measure the fast kinetics of P700^+^, the first 700 ms of the actinic light flash of the first step of the PSI RLC was plotted.

The PQ pool kinetics and *F*_v_/*F*_m_ of the strains were also measured using Dual-PAM 100, with a protocol adapted from Ogawa et al. (2017). The minimal fluorescence of the cells (*F*_0_) was measured in the dark, followed by an induction phase triggered by an illumination period of actinic red light of 200 µmol photons m^−2^ s^−1^ (growth irradiance), which shows the balance of reduction/oxidation of the PQ pool under normal growing light conditions. Samples for this analysis were prepared in the same way as for the PSI ETR. The minimal fluorescence of the cells (*F*_0_) was measured in the dark, followed by an induction phase triggered by an illumination period of actinic far red light of 200 µmol photons m^−2^ s^−1^ (growth irradiance), which shows the balance of reduction/oxidation of the PQ pool under normal growing light conditions. When the cells were in equilibrium, the actinic light was turned off to record the recovery phase. When the PQ pool was fully re-oxidized, the cells were again illuminated with actinic far red light of the same intensity and supplemented with DCMU, to ensure total reduction of the PQ pool. This measurement was used to calculate maximum fluorescence (*F*_m_).

### Protein extraction and quantification

Total protein was extracted from 20 mL of the cryo-preserved cells (see “Culturing conditions”). The cell pellet was resuspended in 300 µL of SDS lysis buffer (200 mM NaCl, 25 mM EDTA, 0.5% [w/v] SDS, 200 mM Tris–Cl, pH 8.5) and then ∼100 mg of 0.1 mm zirconia beads (Biospec Products, Oklahoma, USA) were added. The cells were lysed in a Tissue Lyser (Qiagen, Hilden, Germany) for 3 × 30 s cycles at 30 Hz. The samples were then centrifuged at 12,000 *g* for 10 min at 4°C to pellet the cell debris. The supernatant containing the proteins was removed and then quantified using a BCA assay (Pierce, Thermo Fisher Scientific, Massachusetts, USA) with bovine serum albumin as the standard.

The immunoblotting protocol by Berepiki et al. (2016) was used for relative quantification of CYP1A1. The quantification of PSII and PSI followed the same protocol, with minor changes. In both cases, a total of 2 µg of total protein for each sample were loaded. Where appropriate, PsbA (PSII) and PsaC standards (PSI) were loaded with the samples following the manufacturer’s instructions (Agrisera, Vännäs, Sweden). PsbA and PsaC antibodies (Agrisera, Vännäs, Sweden) were used as primary antibodies for PSII and PSI, respectively. Goat-anti-pAb was used as the secondary antibody for both PSII and PSI. A Versa-Doc imager (BioRad, California, USA) was used for the visualization of the membranes. The proteins were quantified by using the pixel intensity of the bands.

### Transcriptomics—preparation of samples

Cells were collected in triplicate by centrifugation and washed in RNase-free TE buffer (10 mM Tris–HCl, pH 7.5, and 1 mM EDTA). Pelleted cells were reduced to powder with a mortar after freezing in liquid nitrogen. Total RNA was extracted using 1 mL TRIsure reagent (Meridian Biosciences, Ohio, USA) according to the manufacturer’s instructions. Afterward, RNA was extracted with phenol:chloroform (1:1), precipitated with absolute ethanol, and washed with 70% (v/v) ethanol. Finally, RNA was resuspended in 25 µL of RNase-free water. The quality of the RNA was assessed by NanoDrop (Thermo Fisher Scientific, Massachusetts, USA) using the ratios 260/280 and 260/230 nm.

### Transcriptomics—RNA sequencing and bioinformatics

Total RNA extracts were rRNA depleted and RNAseq libraries prepared, using a QIAseq FastSelect rRNA 5S/16S/23S kit (Qiagen, Hilden, Germany) and NEBNext Ultra II Directional RNA Library Prep kit (New England Biolabs, Massachusetts, USA). Individual libraries were indexed using Index Primers from NEBnext Multiplex Oligos for Illumina Sets 1 and 2 (New England Biolabs, Massachusetts, USA); quantified using a Qubit dsDNA HS Assay Kit (Life Technologies, California, USA); quality checked using an Agilent DNA 1,000 Kit (Agilent Technologies, California, USA); then normalized and pooled. Paired end sequencing (2 × 300 bp reads) of the pooled libraries was conducted on the Illumina MiSeq benchtop sequencing platform (Illumina, California, USA) and individual indexed reads were demultiplexed using the Local Run Manager Generate FASTQ Analysis Module (Illumina, California, USA). Library preparation and sequencing was performed by the University of Southampton Environmental Sequencing Facility, and all kits were used in accordance with the manufacturer’s instructions. Resulting raw fastq files were uploaded to the NCBI SRA database, under the BioProject code PRJNA777890.

Bioinformatic analyses were carried out using the Galaxy web platform at usegalaxy.org (Afgan et al., 2018). Raw fastq files were uploaded to the platform, converted to the correct format with FastqGroomer (Blankenberg et al., 2010), and checked for quality with FastQC (http://www.bioinformatics.babraham.ac.uk/projects/fastqc/) Reads were trimmed with Trimmomatic version 0.38 (Bolger et al., 2014) to remove 3′ Illumina adapter sequences (ILLUMINACLIP:TruSeq3-PE.fa:2:30:10) and low-quality 3′ bases (SLIDINGWINDOW:4:15). Trimmed reads were aligned against the complete genome sequence of *Synechococcus* sp. PCC 7002 (NCBI RefSeq accession GCF_000019485.1) using RNA STAR version 2.7.8a (Dobin et al., 2013) and reads aligning to annotated genes were counted using featureCounts from the Subread package version 1.6.4 (Liao et al., 2014) in reverse stranded mode (flag-s 2). Differential gene expression analysis was carried out using DESeq2 (Love et al., 2014). Read counts were modeled using a negative binomial model and pairwise comparisons were carried out to obtain estimates of fold change relative to WT for each condition. Wald tests were used to identify genes showing differential expression between conditions (Benjamini–Hochberg adjusted *P*-value < 0.05).

Gene ontology (GO) enrichment analysis was performed using the TopGO Bioconductor package (Alexa et al., 2006; Huber et al., 2015). GO terms associated with each gene ID were obtained from QuickGO (Binns et al., 2009), using taxID 32049 as a search term. The *Synechoccoccus* genes from QuickGO were annotated with an older locus tag system than the RefSeq assembly that was used as the reference for read alignment, so the RefSeq feature table for GCF_000019485.1 was used to convert between old and new locus tags. For a subset of genes that did not have an old locus tag attribute, GO annotations were obtained using Blast2GO (BioBam Spain, Valencia, Spain). For each pairwise comparison against WT, GO categories that were significantly overrepresented in the differentially expressed genes were identified using the “elim” algorithm in the TopGO package (*P* < 0.05).

### Statistical analyses

All statistical analyses were performed using the R computing environment with base statistics functions. Unless otherwise stated, results are presented as the mean ± sd. Statistical analysis was performed by one-way ANOVA followed by a Tukey’s post hoc test when a significant difference was detected (different letters or asterisks denote statistical significance at *P* < 0.05).

## Accession numbers

Sequence data from this article can be found in the GenBank or Cyanobase under accession numbers: CYP1A1 (NM_012540.2); COX operon (SYNPCC7002_A1162-A1164); *glpK* (SYNPCC7002_A2842); Flv1-3 (SYNPCC7002_A1743/A1321); ARTO (SYNPCC7002_A0725-A0727); and P_*cpcB*_ (genomic sequence 560 bp upstream of the initiation codon of the *cpcB* gene sll1577). All the raw fastq files resulting from the RNA-seq can be found in the NCBI SRA database under the BioProject code: PRJNA777890.

## Supplemental data 

The following materials are available in the online version of this article.

**Supplemental Figure S1.** Growth conditions of the cultures, chlorophyll content, and P450 protein concentration.

**Supplemental Figure S2.** Rapid light curve measurements of PSII and PSI on the studied strains under the inhibitor α-NF.

**Supplemental Figure S3.** PCA plot of the studied strains.

**Supplemental Figure S4.** Venn diagram with the number of genes differentially expressed in the mutant strains.

**Supplemental Figure S5.** Analysis of GO-terms enrichment, identifying cellular functions significantly regulated between cell lines.

**Supplemental Figure S6.** Quantification of PSI:PSII protein ratio via Western blot.

**Supplemental Table S1.** Oligonucleotides used in this work for generating the mutant strains lacking COX.

**Supplemental Table S2.** Fluorescence values acquired during EROD assays under different treatments shown in Figure 2A.

## Supplementary Material

kiac203_Supplementary_Data

## Notes

T.S.B., C.M.M., and M.J.T. conceived the project and were involved in the analysis of the results. A.T. and H.M.C. conducted the work and analyzed the data. A.J.B. and N.P. conducted sequencing and bioinformatics analysis. A.R. constructed some of the strains. A.T. and H.M.C. wrote the manuscript with contributions from all the authors. A.T. agrees to serve as the author responsible for contact and ensures communication.

The author responsible for distribution of materials integral to the findings presented in this article in accordance with the policy described in the Instructions for Authors (https://academic.oup.com/plphys/pages/general-instructions) is: Alejandro Torrado (a.torrado@soton.ac.uk).
