# Supplementary material for: Directing cyanobacterial photosynthesis in a cytochrome c oxidase mutant using a heterologous electron sink
Source: Plant Physiol. 2022 May 6;189(4):2554–66. doi: 10.1093/plphys/kiac203 (PMC9342982; doi:10.1093/plphys/kiac203)
Supplement: kiac203_Supplementary_Data [file kiac203_supplementary_data.pdf]

Supplemental Figure S1

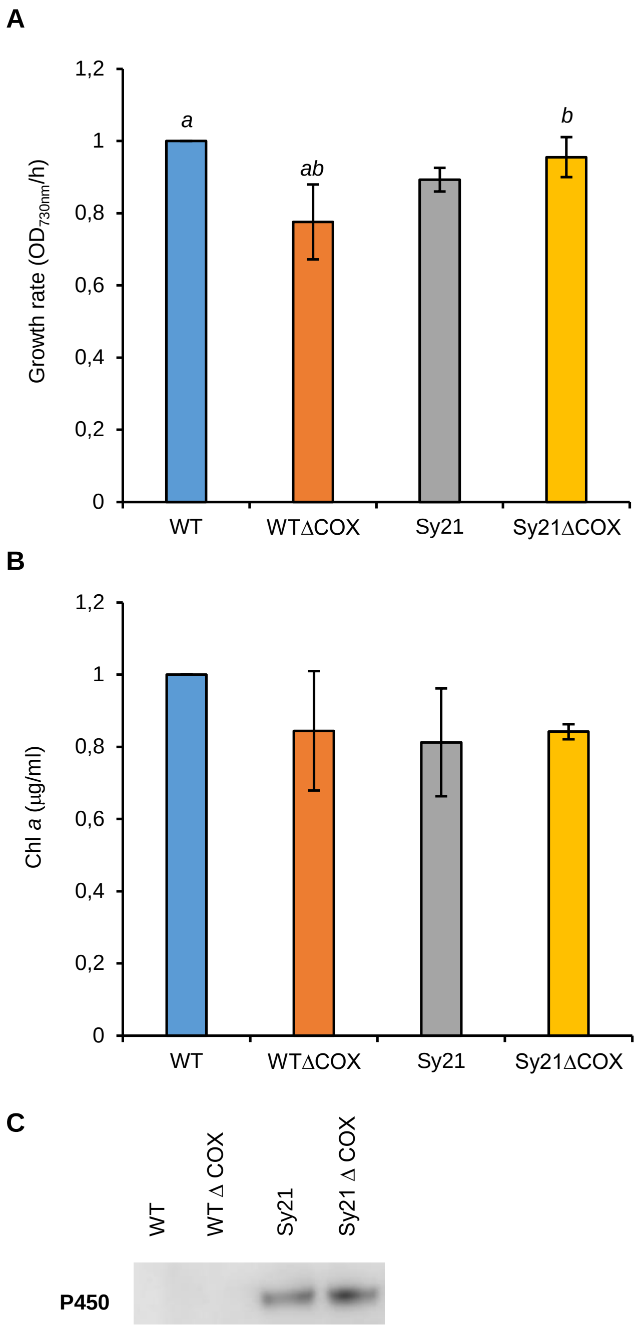

Supplemental Figure S1: Growth conditions of the cultures, chlorophyll content and P450 protein concentration. A) Average growth rate in comparison to WT measured during exponential phase at 24h since inoculation. Each bar is the average of three independent replicates and error bars represent standard deviation of the mean. Different letters indicates significant differences between strains (One way ANOVA, Tukey's post-hoc test, p-value < 0.05). B) Chlorophyll (Chl) *a* concentration in comparison to WT measured during exponential phase at 24h since inoculation. Each bar is the average of three independent replicates and error bars represent standard deviation of the mean. C) Western blot for Cytochrome P450 detection against FLAG antibody. Image shown is a representative of the samples, the actual protein amounts were calculated from three independent replicates, showing no significant difference in P450 quantity between Sy21 and Sy21ΔCOX strains (unpaired two-sample t-test, p-value > 0.05).

Supplemental Figure S2

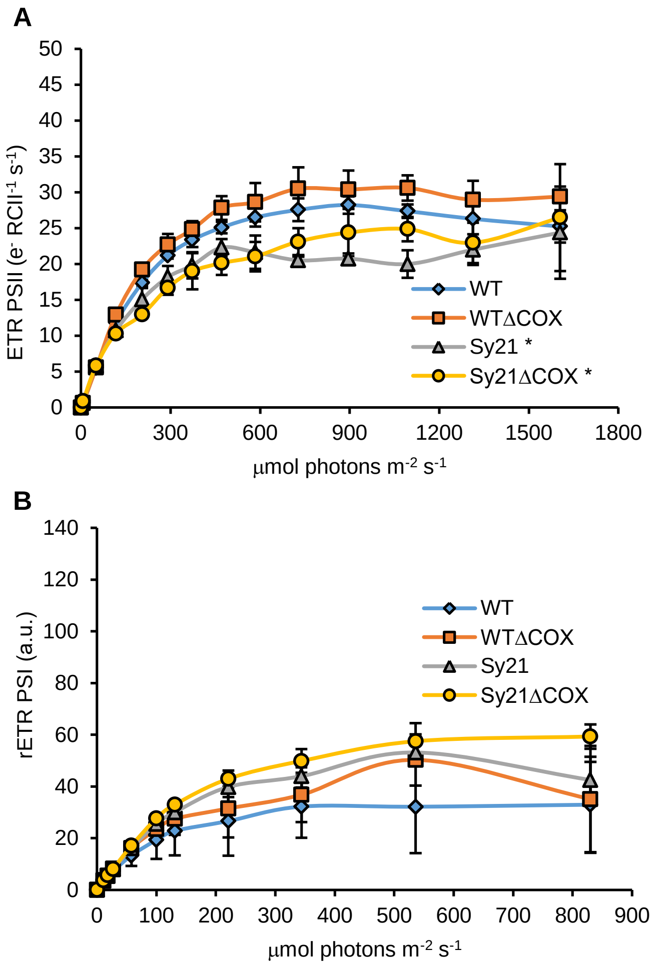

Supplemental Figure S2: Rapid light curve measurements of PSII and PSI on the studied strains under the inhibitor α-naphthoflavone. Rapid light curves describing the number of electrons produced at PSII (A) and PSI (B) at increasing concentrations of light measured in the presence of the P450 inhibitor α-naphthoflavone. Asterisks represent significant differences with WTΔCOX (One-way ANOVA, Tukey's post-hoc test, p-value < 0.05). For the rest of pairwise comparisons of A) and B), no significant differences were found (One way ANOVA, Tukey's post-hoc test, p-value > 0.05). Plotted RLCs are the average produced from three independent replicates; error bars represent standard deviations from the mean. For purpose of comparison, the scale was adjusted to be the same as **Figure 3**.

Supplemental Figure S3

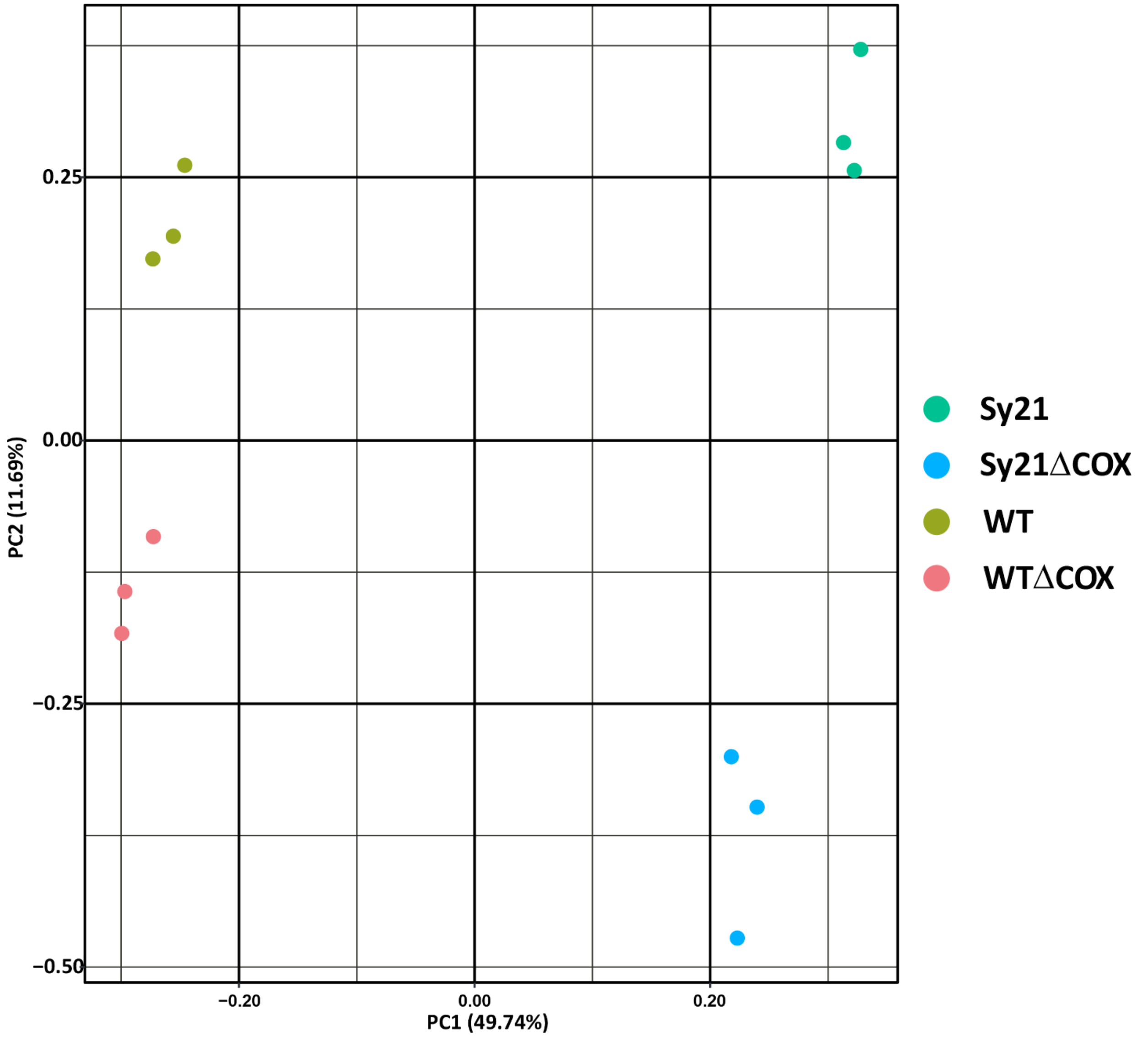

Supplemental Figure S3. PCA plot of the studied strains. The plot was generated from the DESeq2 package, using the usegalaxy platform with standard settings and normal distribution. All strains are represented by three replicates which formed discrete clusters.

Supplemental Figure S4

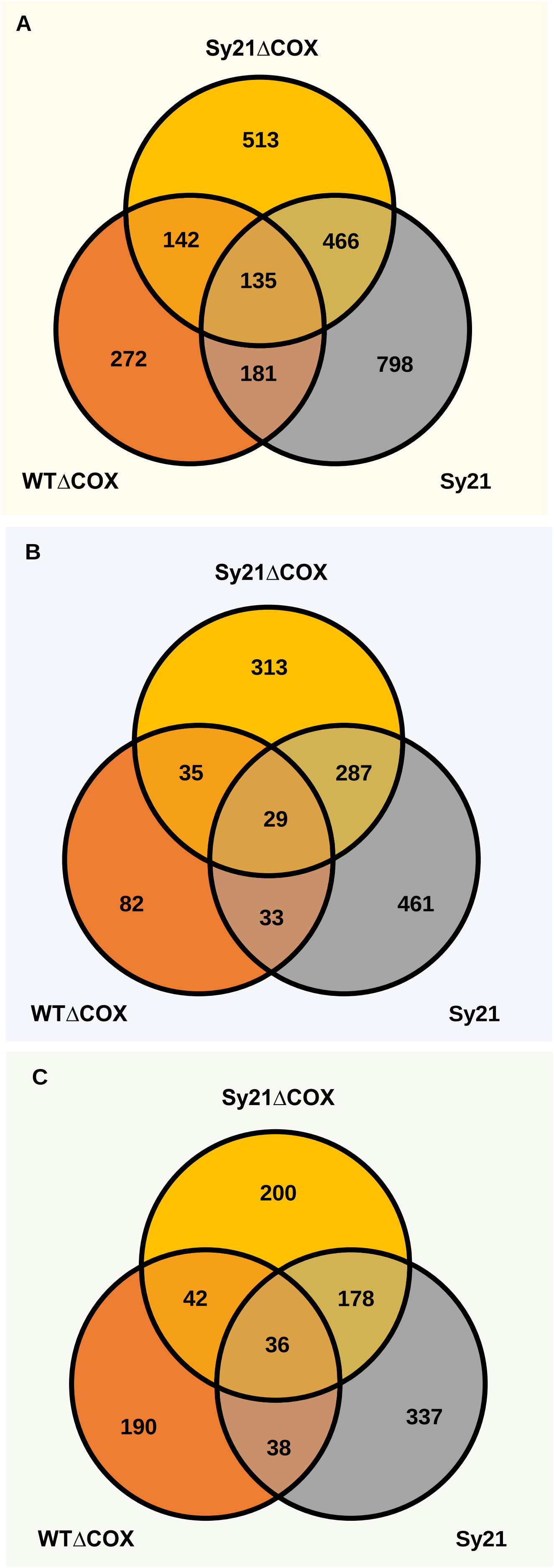

Supplemental Figure S4. Venn diagram with the number of genes differentially expressed in the mutant strains. A) Total numbers of genes differentially expressed in the mutant strains in comparison to WT strain (FDR<0.05); B) and C) show the genes downregulated or upregulated in the mutant strains in comparison to WT, respectively (FDR<0.05).

Supplemental Figure S5

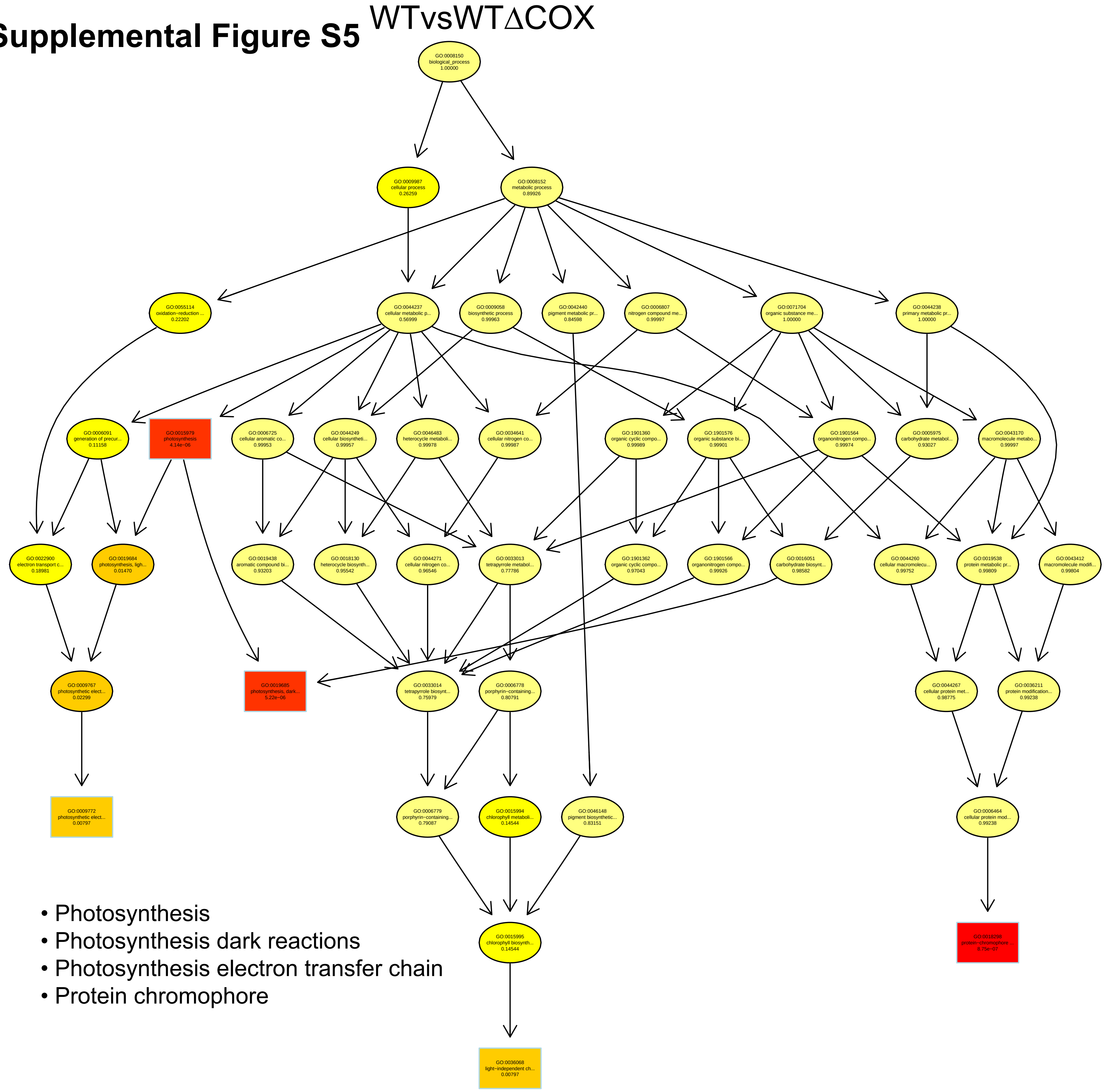

- Photosynthesis
- Photosynthesis dark reactions
- Photosynthesis electron transfer chain
- Protein chromophore

Supplemental Figure S5

WTvsSy21

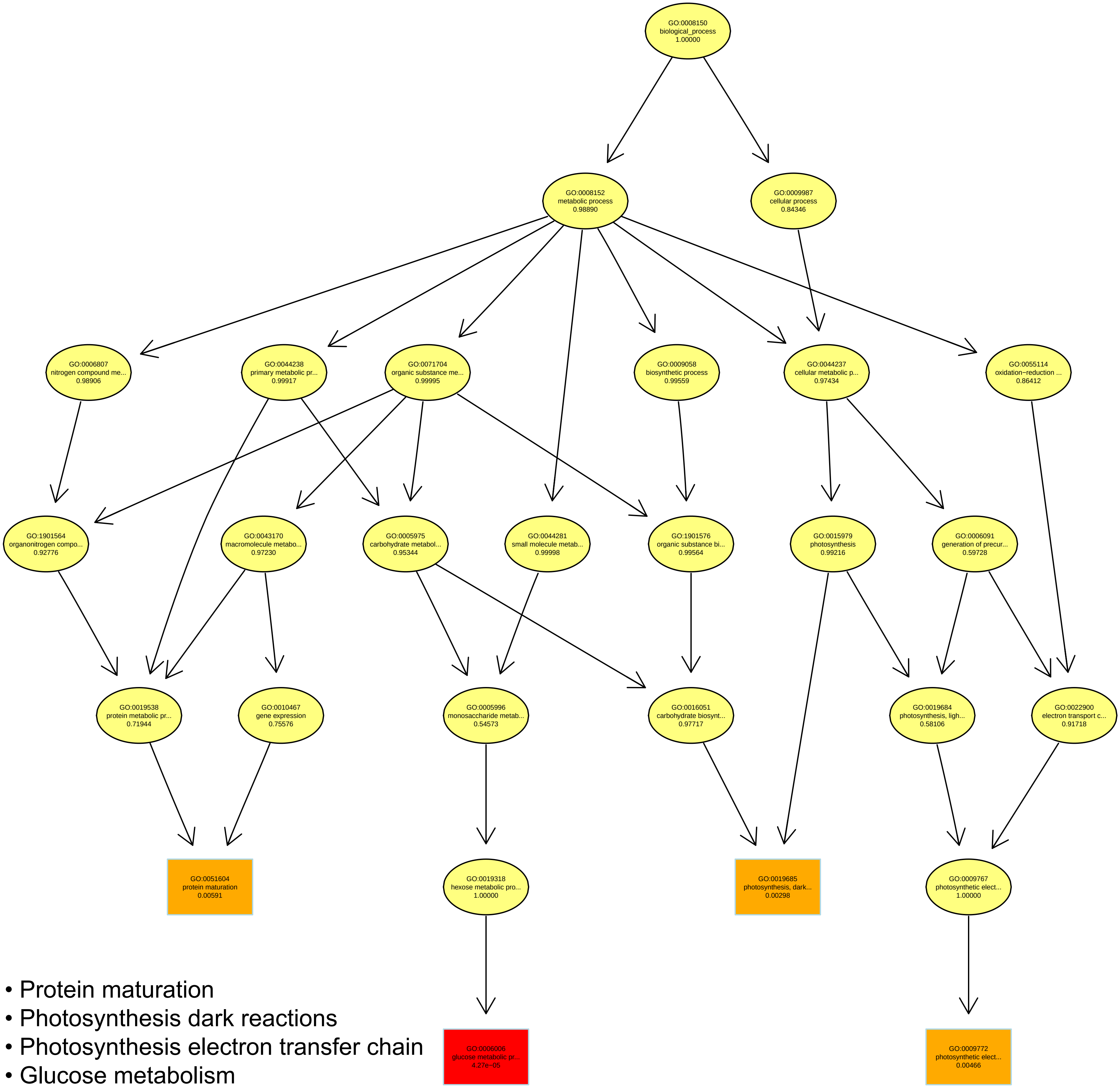

- Protein maturation
- Photosynthesis dark reactions
- Photosynthesis electron transfer chain
- Glucose metabolism

Supplemental Figure S5

WTvsSy21ΔCOX

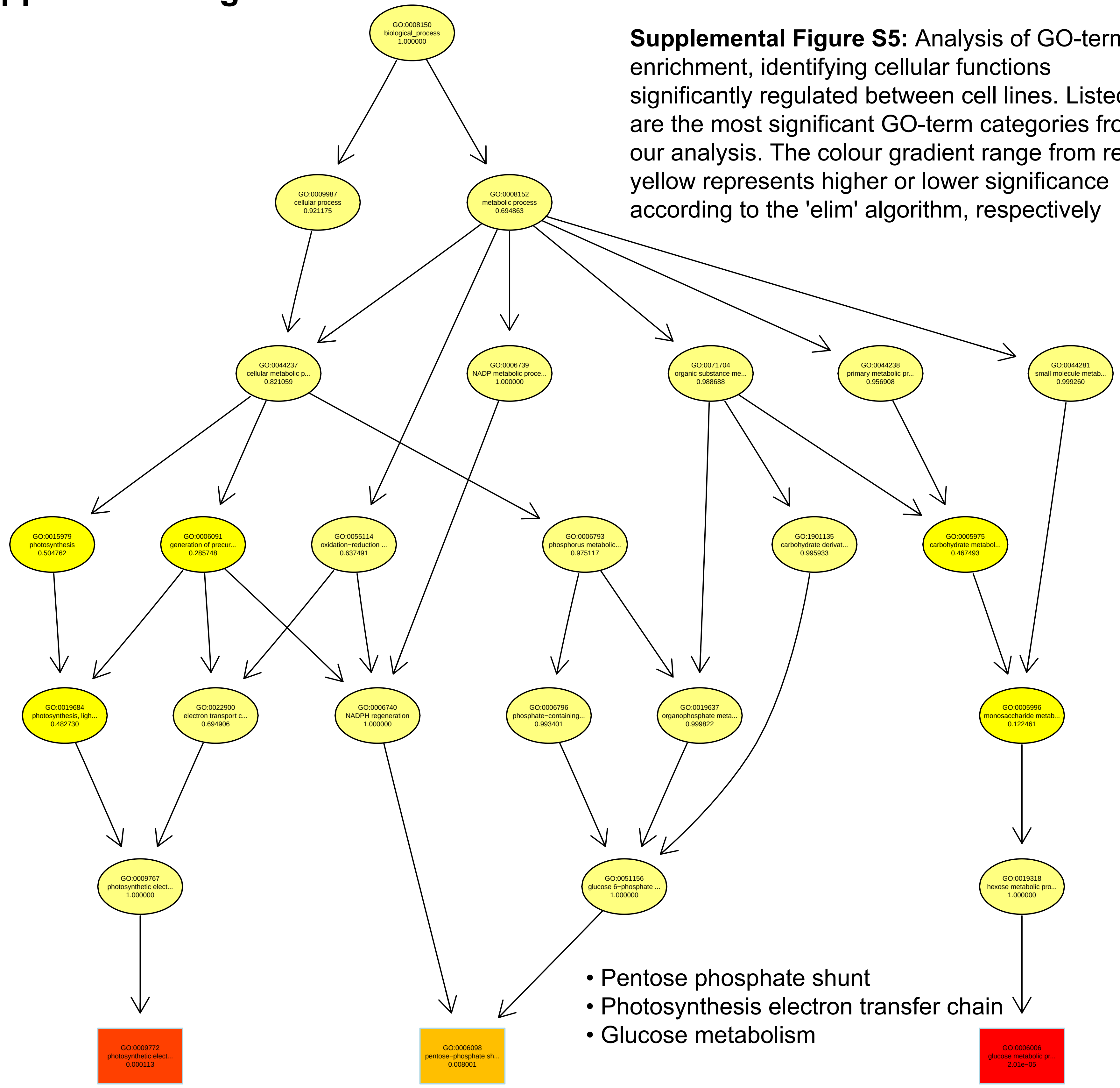

Supplemental Figure S5: Analysis of GO-terms enrichment, identifying cellular functions significantly regulated between cell lines. Listed are the most significant GO-term categories from our analysis. The colour gradient range from red to yellow represents higher or lower significance according to the 'elim' algorithm, respectively

- Pentose phosphate shunt
- Photosynthesis electron transfer chain
- Glucose metabolism

Supplemental Figure S6

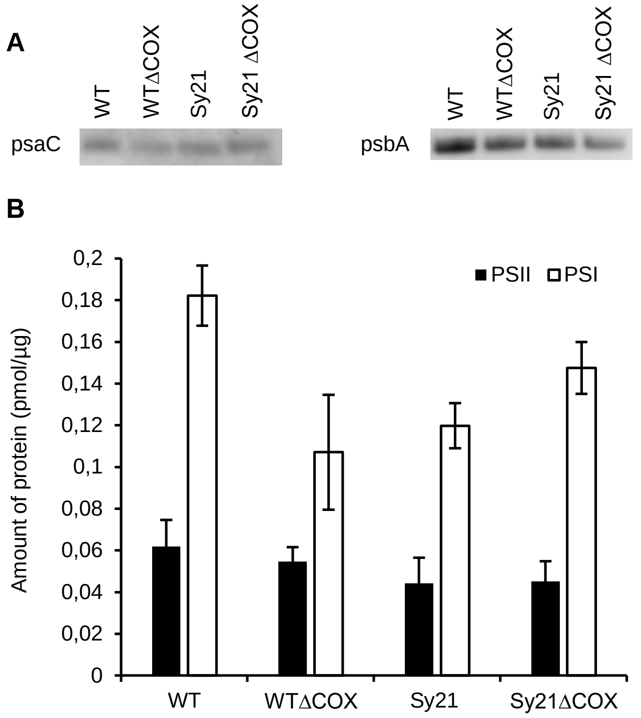

Supplemental Figure S6: Quantification of PSI:PSII protein ratio via Western blot. A) images of the immunoblotted membrane for PSI (PsaC) and PSII (PsbA), 2ng of total protein was loaded for each sample. B) Average PSI protein amount (PsaC) to average PSII protein amount (PsbA), calculated from protein standards in the same blots. Average protein amounts were calculated from three independent replicates. Bars represents standard deviation.

Supplemental Table S1

Name	Sequence 5'-3'
Cox_LF_For	gacgatatcaaagcgtagc
Cox_LF_Rev	gaaatgttggagcttttttgg
Gen_For	ccaaaaaagctccaacatttccgaatccatgtgggagttta
Gen_Rev	gaaatgaagctaTTACAGCAGATAttaggtggcggta cttgggt
Cox_RF_For	TATCTGCTGTAAtagcttcatttc
Cox_RF_Rev	ccattattttccgcctacg

Supplemental Table S1. Oligonucleotides used in this work for generating the mutant strains lacking COX.

Supplemental Table S2

Strain	Control Fluorescence (a.u.)	α-NF Fluorescence (a.u.)	DCMU Fluorescence (a.u.)
Blank	3936	3809	3972
WT	3845	3713	3914
WTΔCOX	3796	3824	3886
Sy21	5219	4000	4256
Sy21ΔCOX	9165	4226	5107

Supplemental Table S2. Fluorescence values acquired during EROD assays under different treatments shown in Figure 2A. Each value is the mean of three independent replicates. Blank: negative control with media; α-NF: α-naphtoflavone; DCMU: 3-(3,4-dichlorophenyl)-1,1-dimethylurea.
